# Impact of postoperative complications on long-term survival following surgery for T4 colorectal cancer

**DOI:** 10.1186/s12893-018-0419-y

**Published:** 2018-10-17

**Authors:** Michael Osseis, Francesco Esposito, Chetana Lim, Alexandre Doussot, Eylon Lahat, Liliana Fuentes, Toufic Moussallem, Chady Salloum, Daniel Azoulay

**Affiliations:** 1 Department of Digestive, Hepatobiliary and Pancreatic Surgery and Liver Transplantation, Henri Mondor Hospital, APHP, UPEC, 51 avenue de Lattre de Tassigny, 94010 Créteil, France; 2 INSERM, U955, Créteil, France

**Keywords:** Postoperative complications, Colorectal, Survival, Recurrence, T4 tumors

## Abstract

**Background:**

Postoperative complications (POCs) after the resection of locally advanced colorectal cancer (CRC) may influence adjuvant treatment timing, outcomes, and survival. This study aimed to evaluate the impact of POCs on long-term outcomes in patients surgically treated for T4 CRC.

**Methods:**

All consecutive patients who underwent the resection of T4 CRC at a single centre from 2004 to 2013 were retrospectively analysed from a prospectively maintained database. POCs were assessed using the Clavien-Dindo classification. Patients who developed POCs were compared with those who did not in terms of recurrence-free survival (RFS) and overall survival (OS).

**Results:**

The study population comprised 106 patients, including 79 (74.5%) with synchronous distant metastases. Overall, 46 patients (43%) developed at least one POC during the hospital stay, and of those patients, 9 (20%) had severe complications (Clavien-Dindo ≥ grade III). POCs were not associated with OS (65% with POCs vs. 69% without POCs; *p* = 0.72) or RFS (58% with POCs vs. 70% without POCs; *p* = 0.37). Similarly, POCs did not affect OS or RFS in patients who had synchronous metastases at diagnosis compared with those who did not.

**Conclusions:**

POCs do not affect the oncological course of patients subjected to the resection of T4 CRC, even in cases of synchronous metastases.

**Electronic supplementary material:**

The online version of this article (10.1186/s12893-018-0419-y) contains supplementary material, which is available to authorized users.

## Background

Colorectal cancer (CRC) is the third most common cancer type and the fourth cause of cancer-related deaths worldwide [1]. Nearly 10% to 20% of patients with CRC present with locally advanced disease, including peritoneal involvement (T4a) or invasion in adjacent organs (T4b) at diagnosis [2].

Long-term survival has improved in selected patients with clinically T4 colorectal cancer managed with the multimodal treatment strategy including surgery and perioperative chemotherapy. Patient selection remains of utmost importance as CRC resection for T4 lesions is associated with significant morbidity rate ranging from 30 to 40% [3, 4].

Postoperative complications are associated with increased hospital stay and in-hospital costs [5, 6]. There is increasing evidence reporting that the postoperative complications were also risk factors for the survival or tumor recurrence in various types of abdominal malignancies including esophageal, gastric, and liver cancers [7–10]. In CRC patients, reports that have studied the effect of postoperative morbidity following resection on long-term survival have yielded conflicting results. To our knowledge, the effect of postoperative morbidity following surgery of T4 CRC has never been reported. Despite several studies performed in CRC patients, most studies have many limitations including heterogeneous disease stages populations, with a relatively small sample size of T4 CRC patients (Table 1).

**Table 1:** Literature regarding the influence of morbidity on long-term survival after resection of colorectal cancer from 2004 to 2017

1st Author (reference), year, country	Study period	Duration of FU (months)	Compared morbidity	Number of patients	% of T4 (n)	Post-op complications (%)	AL rate (%)	Impact of morbidity on OS (at 5 years)	Location of cancer	Independent predictor of OS hazard ratio
Law et al. [31] 2004, Hong Kong	1993–2002	40 (3–109)^a^	AL versus No AL	563	NA	6	6	8/30 vs 156/533, *p* = 0.7600	R	*p* = 0.004
Eriksen et al. [32] 2004, Norway	1993–1995	45 (0–98)^a^	AL versus No AL	1958	6 (117/1958)	NA	22 (T4)	93/228 vs 564/1730, *p* = 0.014	R	NA
Walker et al. [33] 2004, Australia	1971–1999	≥60^a^	AL versus No AL	1722	NA	NA	5	44.3% vs 64%, *p* = 0.0001	R/C	1.6 (CI 1.3–2.1)
Nespoli et al. [34] 2004, Italy		≥60^a^	Yes, versus No	192	NA	NA	NA	*p* = 0.0006	C	NA
Branagan and Finnis et al. [35] 2005, UK	1991–1995	≥60^a^	AL versus No AL	1759	NA	NA	4	R: 17/36 vs 210/581, *p* = 0.1840 C: 9/22 vs 430/112, *p* = 0.8100	R/C	NA
McArdle et al. [36] 2005, UK	1991–1994	≥60^a^	AL versus No AL	2235	NA	NA	4	43/86 vs 688/2149, *p* = 0.0010	R/C	1·61 (CI 1·19, 2·16)
Ptok et al. [37] 2007, Germany	2000–2001	≥40^a^	AL versus No AL	1625		50 for (AL group) 26 (for Non-AL group)	15	74/255 vs 337/1370, *p* = 0.1360	R	NA
Law et al. [22] 2007, Hong Kong	1996–2004	46^c^	Yes, versus No	1657	NA	27	4	26/60 vs 366/1520, *p* = 0.0010	R/C	1.26, *p* = 0.023
Lee et al. [38] 2008, Korea	1996–2006	44.6 (2–157)^a^	AL versus No AL	1278	73 (933/1278) (T3/T4)	NA	4	80.2% vs 64.9%, *p* = 0.17	R	NA
Eberhardt et al. [34] 2009, USA	1979–2007	≥60^a^	AL versus No AL	468	NA	NA	33	13/59 vs 16/118, *p* = 0.1550	R/C	NA
Den Dulk et al. [39] 2009, Multinational	1987–2002	70.8 (2–179)^a^	AL versus No AL	2726	NA	NA	10	46/220 vs 427/2199, *p* = 0.5950	R	1.48
Bertelsen et al. [40] 2009, Denmark	2001–2004	45.2 (1–74)^a^	AL versus No AL	1494	NA	NA	11	50/357 vs 95/1077, *p* = 0.005	R	1.63 (CI 1.21–2.19)
Jogren et al. [41] 2009, Sweden	1995–1997	60^c^	AL versus No AL	250	10 (25/250)	NA	NA	63% vs 66%, *p* = 0.38	R	1
Mirnezami et al. [42] 2011, UK	1965–2009	–	AL versus No AL	21,902	–	–	–		R/C	1.75 (CI 1.47–2.1), *p* = 0.0001
Gooiker et al. [43] 2012, Netherland	2006–2008	48^c^	Yes, versus No	2131	NA	20	NA	12% vs 26%, *p* = 0.02 (1 year)	R/C	5.9 (CI 1.3–26.8)
Smith et al. [28] 2013, USA	1992–2010	35^c^	AL versus No AL	184	7		11	32 vs 72%, *p* = 0.01	R	NA
Henneman et al. [44] 2014, Netherland	2011–2012	NA	Yes, versus No	10,184	NA	NA	C: 27 R: 36.5	NA	R/C	NA
Odermatt et al. [45] 2015, UK	2003–2012	≥60^a^	AL versus No AL	844	10 (84/844)	5	3	*p* = 0.80	R/C	1.04 (CI 0.76–1.42)
Ebinger et al. [46] 2015, Germany	1991–2010	62 (2–254)^a^	AL versus No AL	584	NA	NA	11	*p* = 0.701	R	0.91 (CI 0.56–1.47)
Kulu et al. [30] 2015, Switzerland	2002–2011	56 ± 35^b^	AL versus No AL	570	NA	NA	9	65% vs 83%	R	2.27 (CI 1.33–3.88), *p* = 0.005
Hain et al. [29] 2016, France	2005–2014	40 ± 27^b^	AL versus No AL	428	50 (214/428) (pT3/T4)	50	28	78·6% vs 88·4%, *p* = 0.001	R	NA
Breugom et al. [47] 2016, Netherland	2006–2008	64.8 (56.4–74.4)^a^	Yes, versus No	761	75 (570/761) (T3/T4)	42	5	65% vs 35%, *p* = 0.001	C	1.59 (CI 1.25–2.04), *p* = 0.001
Jamnagerwalla et al. [48] 2016, Australia	2003–2014	46^c^	Chemotherapy versus No Chemotherapy	517	100 (517/517) (T3/T4)	28	3.5	NA	R	0.53, *p* = 0.04
Nordholm-Carstensen et al. [49] 2017, Denmark	2009–2013	37 (25–50)^a^	Yes, versus No	774	NA	NA	9	C: 18.7% vs 44.6% *p* < 0.001 R: 53.7% vs 73.3% *p* = 0.0600	R/C	C: 1.67 (CI 1.03–2.68), *p* = 0.04 R: 0.93 (CI 0.24–3.57), *p* = 0.91
Present study 2017, France	2004–2013	42	Yes, versus No	106	100 (106/106)	43	4	65% vs. 69%; *p* = 0.72	R/C	*p* > 0.05

Footnotes: FU indicates follow-up; *AL* anastomotic leakage, *OS* overall survival, *NA* not available, *R* rectum, *C* Colon, *CI* confidence interval

^a^No mean or median given

^a^Median given with range. ^b^Mean given with range. ^c^Median given with no range

The above knowledge was the impetus for the present study which aimed to evaluate the impact of postoperative morbidity on long-term outcomes following potentially curative resection for colorectal cancer.

## Methods

### Patients

All consecutive patients who underwent either elective or urgent surgery with histologically proven T4 CRC on final resected specimens were retrospectively identified from a prospectively maintained database of patients undergoing laparoscopic or open colorectal resection at Henri Mondor Hospital between January 2004 and December 2013. Patients were categorized into two groups: with synchronous distant metastases (stage IV according to the American Joint Committee on Cancer) or without (stage II–III). Patients who died within 90 days of surgery were not considered for inclusion in this study because they were not exposed to recurrence.

### Perioperative management and surgical techniques

All patients underwent a preoperative evaluation, including colonoscopy with tumour biopsy and thoraco-abdominopelvic computed tomography (CT) scan. In cases of rectal cancer, pelvic magnetic resonance imaging (MRI) and endorectal ultrasonography were performed for local rectal cancer staging. Liver MRI was systematically performed in case of synchronous liver metastases diagnosed by CT.

In patients with mid or low rectal cancer who underwent elective surgery, the indications were for neoadjuvant long-course chemoradiation therapy (45–50.4 Gy delivered in daily fractions of 1.8–2 Gy over a 5- to 6-week period combined with 5-fluorouracil [5-FU] or capecitabine [Xeloda]). Short-course radiotherapy (5 × 5 Gy for 1 week) or chemotherapy alone were determined by multidisciplinary cancer boards according to local standards. Surgery was performed 6 to 8 weeks after the completion of chemo-radiotherapy and immediately after short-course radiotherapy.

All patients were operated with a curative intent. During the study period, the following oncological principles were applied: vascular control at the root of the corresponding mesenteric axis for appropriate lymphadenectomy and multivisceral en bloc resection in cases of adhesion to adjacent organs. Total mesorectal excision was performed in cases of mid or low rectal cancer [11]. Curative resection was defined as the complete removal of all macroscopically evident disease at the time of surgery and tumour-free resection margins on histological examination. A diverting ileostomy was performed in all cases of infraperitoneal colorectal anastomosis.

### Definitions and study design

Any postoperative event occurring within 90 days and deemed as leading to any deviation from the normal postoperative course was considered a complication [12]. Surgical complications included anastomotic leakage, bleeding, ileus, intraabdominal or pelvic abscess, and wound infection. Anastomotic leakage was defined and given one of three grades (A, B and C) according to the international study group of rectal cancer [13]. Non-surgical complications included renal, pulmonary, cardiac, and infectious complications. Postoperative complications (POCs) were graded according to the Clavien-Dindo staging system [14]. Grade III and IV complications were considered as severe complications.

All patients participated in an oncological follow-up program every 3 months for the first 2 years and every 6 months thereafter. Abdominal and chest CT scans with a blood test including carcinoembryonic antigen levels were routinely performed during every follow-up visit. A full colonoscopy was performed 1 to 2 years after surgery and then once every 4 years. If recurrence was suspected, MRI and/or positron emission tomography-CT were used to confirm the diagnosis. Biopsies were selectively performed.

Patients were divided in two groups: patients who did and did not develop POCs. The two groups were then compared in terms of OS and recurrence-free survival (RFS). Additionally, the time from surgery to adjuvant chemotherapy was retrieved to measure the impact of POCs on adjuvant chemotherapy delivery.

This study was approved by the local institutional review board and ethics committee of Henri Mondor Hospital, conformed to the ethical guidelines of the 1975 Declaration of Helsinki.

### Statistical analysis

Continuous variables are presented as the mean (SD); all other variables are presented as the median (range) and were compared using the Mann-Whitney U test. RFS and OS were estimated using the Kaplan-Meier method. Survival differences between groups were compared using the log-rank test. Variables that reached statistical significance (*p* < 0.05) in univariate analyses were included in a Cox proportional hazard model to identify independent prognostic predictors of OS and RFS. All analyses were performed using SPSS® version 22.0 (IBM, Armonk, New York, USA).

## Results

### Study population

According to the objective of the study, 17 patients were excluded from the analysis—8 (6.5%) died within 90 days of surgery, and 9 were lost to follow-up. The remaining 106 patients represented the study population (Table 2). The tumour was rectal in 15 patients (14%) and colonic in 92 patients (86%). One patient had combined colon and rectal cancer. Six patients (5.6%) underwent preoperative and postoperative chemo-radiotherapy, and 10 patients (9%) underwent preoperative chemotherapy alone. At presentation, 27 (26%) patients had synchronous metastases: 18 (67%) had stage IVA (liver only), 4 (15%) had stage IVA (lung only), and 5 (19%) had stage IVB CRC. Eighty-six patients (81%) underwent elective CRC resection, and 20 (19%) patients underwent emergent resection due to perforation or bleeding.

**Table 2:** Demographics, perioperative variables, and histopathological findings

Variable	Total *n* = 106	No complication *n* = 60 (56%)	Any complication *n* = 46 (44%)	*P*
Age (years)	69 ± 14	70 ± 14	67 ± 14	0.27
Male sex	46 (43%)	25 (42%)	21 (46%)	0.68
ASA score > 2	13 (12%)	8 (61.5%)	5 (38.5%)	0.70
BMI	24 ± 6	24 ± 6	25 ± 6	0.32
Comorbidity				
Cardiovascular	45 (42%)	25 (55.5%)	20 (44.5%)	0.85
Pulmonary	20 (19%)	6 (30%)	14 (70%)	0.01
Diabetes	17 (16%)	11 (65%)	6 (35%)	0.46
Localization				
Rectum	14 (13%)	9 (64%)	5 (36%)	0.53
Colon	92 (86%)	51 (55%)	41 (45%)	
Synchronous metastasis	27 (25%)	15 (55.5%)	12 (44.5%)	0.90
Stage IVA (liver only)	18 (67%)	9 (50%)	9 (50%)	
Stage IVA (lung only)	4 (15%)	3 (75%)	1 (25%)	
Stage IVB	5 (19%)	3 (60%)	2 (40%)	
Serum CEA (μ/L)	54 ± 116	71 ± 140	37 ± 82	0.25
Neoadjuvant radiotherapy or chemotherapy	16 (15%)	9 (56%)	7 (44%)	0.98
Operative setting				0.51
Elective	84 (79%)	48 (57%)	36 (43%)	
Emergent	22 (21%)	12 (54.5%)	10 (45.5%)	
Surgical procedure				0.85
Abdominoperineal resection	2 (1.8%)	1 (50%)	1 (50%)	
Hartmann’s procedure	7 (6.6%)	3 (43%)	4 (57%)	
Anterior resection	17 (16%)	11 (65%)	6 (35%)	
Segmental resection	80 (75.5%)	45 (56%)	35 (44%)	
Surgical approach				0.75
Open	78 (74%)	43 (55%)	35 (45%)	
Laparoscopic	28 (26%)	11 (39%)	17 (61%)	
Associated resection	40 (37.8%)	23 (57.5%)	17 (42.5%)	
1 organ	23 (49%)	15 (65%)	8 (35%)	0.51
> 1 organ	17 (51%)	8 (47%)	9 (53%)	
Synchronous liver resection	11 (10.3%)	5 (45%)	6 (55%)	0.88
Stoma	42 (39.6%)	21 (50%)	21 (50%)	0.27
Specimen analysis N+	60 (56.6%)	35 (58%)	25 (42%)	0.68
Surgical margins status				0.35
R0	85 (80%)	35 (41%)	50 (59%)	
R1	21 (20%)	11 (52%)	10 (48%)	
Adjuvant chemotherapy	65 (61%)	32 (53%)	33 (72%)	0.06
Delay from surgery to chemotherapy (days)	52 ± 50	55 ± 62	49 ± 33	0.69

Footnotes: *ASA* American Society of Anaesthesiologists, *BMI* body mass index, *CEA* carcinoembryonic antigen

### Perioperative data and specimen analysis

Intraoperative data are reported in Table 2. Surgery was performed by an open approach in 78 (74%) patients and by a laparoscopic approach in 28 (26%) patients. The surgical procedures included segmental colectomy in 80 patients (75%), low anterior resection of the rectum in 17 patients (16%), Hartmann’s procedure in 7 patients (6.6%), and abdominoperineal resection in 2 patients (1.9%). Temporary faecal diversion was performed in 42 patients (40%). In the latter subset of patients, the cancer was rectal in 13 cases, left colonic in 16 cases, right colonic in 9 cases, and transverse in 4 cases. Resection of adjacent organs was needed in 40 patients (38%): one organ in 23 patients (22%) and more than one organ in 17 patients (16%). Concomitant hepatectomy for synchronous liver metastases was performed in 11 patients (10%), and the en bloc resection of organs adjacent to the tumour was required in 29 (27%) patients. The number and types of additional organs resection are reported in Additional file 1: Table S1.

Pathological findings included 21 R1 (20%) and 85 R0 (80%) CRC resections. The malignant infiltration of adherent organs was observed in 22 patients (21%). Lymph nodes that tested positive for disease were found in 60 patients (57%).

### Post-operative complications

Pre- and perioperative variables associated with the development of POCs are presented in Table 2. Globally, the two groups did not differ in terms of demographics, clinical, and perioperative outcomes.

Forty-six patients developed POCs (morbidity rate = 43%), and the rate of severe complications (Clavien-Dindo grade ≥ 3) was 8.5% (9 patients). Four patients (3.7%) had anastomotic leakage—two were classified as grade A anastomotic leakage, and the other two were considered grade B. Details of complications are detailed in Table 3.

**Table 3:** Details of postoperative complications among 106 patients

	No. of patients (%)
Anastomotic leakage	4 (3.7%)
Infectious complications	
Pelvic abscess	6 (5.6%)
Intra-abdominal abscess	6 (5.6%)
Urinary infection	4 (3.7%)
Wound infection	10 (9.4%)
Non-infectious complications	
Ileus	6 (5.6%)
Kidney failure	2 (1.8%)
Pulmonary failure/pleuresia	4 (3.7%)
Intra-abdominal bleeding	1 (0.9%)
Cardiac problems	2 (1.8%)

According to Clavien-Dindo classification

Patients may have had more than one complication

### Adjuvant therapy

Sixty-five patients (61%) received adjuvant chemotherapy after surgery. There was no significant difference in the delivery of adjuvant chemotherapy between the patient groups with and without POCs (53% vs. 72%, respectively; *p* = 0.06). The delay from surgery to chemotherapy was not different between the two groups (55 vs. 49 days, respectively; *p* = 0.69).

### Impact of POCs on long-term outcomes

#### All stages combined

**Fig. 1:**
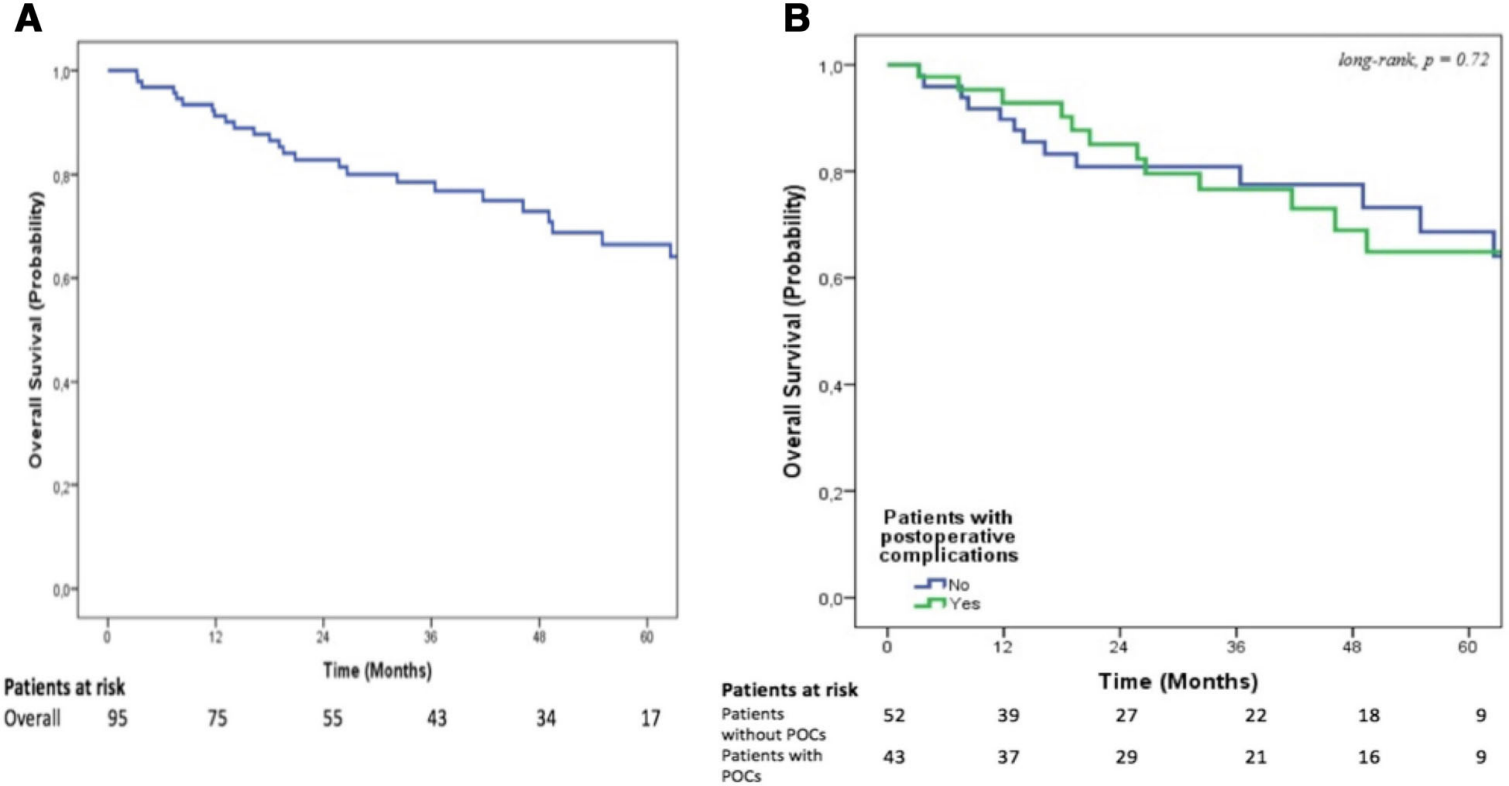
Overall survival. **a** In the entire cohort. **b** Stratified according to the presence of postoperative complications

**Fig. 2:**
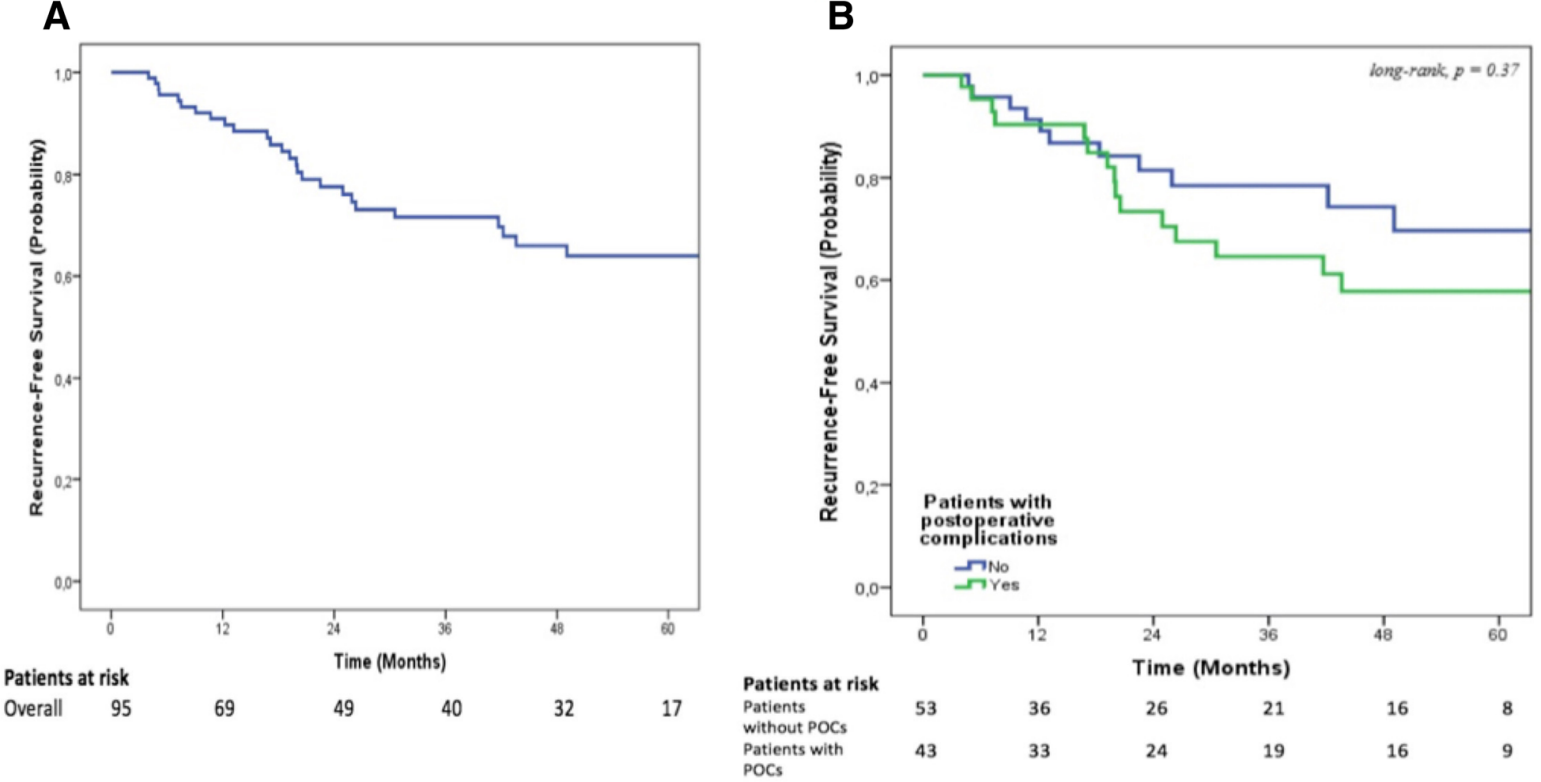
Recurrence-free survival. **a** In the entire cohort. **b** Stratified according to the presence of postoperative complications

The median follow-up was 42 [4–125] months. Overall, the 1-, 3- and 5-year OS rates were 91%, 79%, and 67%, respectively (Fig. 1). The 1-, 3- and 5-year RFS rates were 91%, 72%, and 64%, respectively (Fig. 2). In the multivariable analysis, no variables were identified as predictors of OS (data not shown), while the presence of positive lymph nodes was the sole independent predictor of decreased RFS rate (Table 4). POCs did not impact either OS or RFS in the entire cohort.

**Table 4:** Uni- and multivariate analyses of risk factors for overall and recurrence-free survival in the entire cohort (*n* = 106)

Variable	Univariate *P* value	Multivariate *P* value	Hazard Ratio (95% Confidence Interval)
Age ≥ 60 (years)	0.76		
Male sex	0.37		
BMI ≥ 30 (kg/m^2^)	0.79		
ASA score ≥ 2	0.12		
Elevated CEA	0.03	0.46	
Colon vs rectum	0.59		
Synchronous metastases	0.03	0.21	
Neoadjuvant treatment	0.45		
Emergent surgery	0.46		
Laparoscopic approach	0.51		
Multiple organ resection	0.88		
Synchronous liver resection	0.43		
N+ status	0.009	0.01	3 (1–7)
R1 margins	0.03	0.08	
Postoperative complications	0.37		
Grade III-IV complications	0.57		
Adjuvant chemotherapy	0.80		

Footnotes: *BMI* indicates body mass index, *ASA* American Society of Anesthesiologists, *CEA* carcinoembryonic antigen

#### Stage I–III disease

In patients without synchronous metastases, the 1-, 3- and 5-year OS rates did not differ between the two groups (94%, 80%, and 75%, respectively, in the POCs group vs. 91.4%, 82%, and 74.6%, respectively, in the no POCs group; *p* = 0.77). In the multivariable analyses, no variables were identified as predictors of OS; however, three variables were identified as independent predictors of low RFS rates: ASA score > 2, positive lymph nodes, and R1 margins (Table 5). POCs did not impact either OS or RFS in patients who did not have synchronous metastases.

**Table 5:** Uni- and multivariate analyses of risk factors for overall and recurrence-free survival in patients without synchronous metastases (*n* = 79)

Variable	Univariate P Value	Multivariate P Value	Hazard Ratio (95% Confidence Interval)
Age ≥ 60 years	0.39		
Male sex	0.28		
BMI ≥ 30 kg/m2	0.79		
ASA ≥ 2	0.03	0.03	4 (1–13)
Elevated CEA	0.17		
Colon vs rectum	0.77		
Synchronous metastases	–		
Neoadjuvant treatment	0.17		
Emergent surgery	0.55		
Laparoscopic approach	0.96		
Multiple organ resection	0.81		
Synchronous liver resection	–		
N+ status	0.03	0.01	4 (1–13)
R1 margins	0.05	0.02	3 (1–8)
Postoperative complications	0.90		
Grade III-IV complications	0.82		
Adjuvant chemotherapy	0.70		

Footnotes: *BMI* indicates body mass index, *ASA* American Score of Anesthesiologists, *CEA* carcinoembryonic antigen

#### Stage IV disease

In patients with synchronous metastases, the 1-, 3- and 5-year OS rates did not differ between the two groups (90%, 65.6%, and 21.9%, respectively, in the POCs group vs. 85.7%, 77.9%, and 59.4%, respectively, in the no POCs group; *p* = 0.35).

## Discussion

To our knowledge, this is the first study to evaluate the effect of POCs on long-term outcomes following resection of T4 CRC. In this present single centre analysis of a homogeneous group of consecutive T4 CRC patients, OS and RFS rates were not significantly different between patients who developed POCs and those who did not. These results were maintained after patients’ stratification for the presence of synchronous metastases.

In the present study, the overall morbidity rate was 43%. This is consistent with the values reported in recent reports (POCs in the range of 33–45%) [4, 15].

The laparoscopic approach was used in a relatively low proportion of patients in our study (26%). Although the impact of this approach on postoperative morbidity and survival was beyond the scope of this study, the laparoscopic approach might contribute to contain the postoperative morbidity (POCs in the range of 7–26%) [16–18] and to improve the oncologic results [16]. These results were further confirmed by two recent studies using propensity score methodology [19, 20]. However, the rate of conversion rate remains relatively high, varying between 8 and 28% [16–18, 21]. Further studies are needed to ascertain the real impact, if any, of laparoscopic approach on the incidence of postoperative morbidity in the specific setting of T4 CRC.

In this study, POCs did not impact on OS. The impact of POCs on the long-term prognosis of patients following different surgeries has recently been investigated. Khuri et al. used data from the National Surgical Quality Improvement Program to study the effects of POCs on the survival rate of more than 100,000 patients who underwent eight major operations [7]. In contrast with our results, the study showed that the occurrence of POCs within the first 30 days, independent of the patient’s preoperative risk, reduced the median survival by 69%. This latter study also showed that in patients who underwent a colectomy, there was a significant difference of 14.5% in mortality at 5 years between those who did and did not have complications. However, it is important to note that the group of patients who underwent colectomies in this study (13,310/100,000 patients) is a heterogeneous group with different indications for colectomy, not only for colon cancer. The present study included only patients who underwent surgical treatment for locally advanced CRC, which might explain this discrepancy between the results of the 2 studies. In a recent study by Law et al. [22], the occurrence of POCs was an independent factor associated with a worse overall survival and a higher overall recurrence rate. However, the impact of POCs on the survival and oncologic outcome in patients with T4 CRC was not clarified.

Whereas positive lymph node status was identified as the sole independent predictor of a decreased RFS, POCs did not impact on DFS even after patients’ stratification for the presence of synchronous distant metastases. These findings are in agreement with previous reports [23, 24]. Based on this, it could be argued that tumor biology rather than postoperative morbidity remained the main determinant of survival in these patients.

The debate regarding whether POCS may delay the initiation of adjuvant chemotherapy after surgery remains active [25, 26]. The present study showed that patients who developed POCs had similar delay in time to adjuvant chemotherapy than those who did not (*p* = 0.69). However, the relatively high rate of adjuvant chemotherapy (72%) in the POCs group might explain the similar long-term outcome between these 2 groups. As reported in a recent study in the field of pancreatic cancer surgery, a minimally invasive surgery approach may offer earlier time to adjuvant chemotherapy [27]. Further studies are required to assess the potential impact of minimally invasive surgery on the delay to adjuvant chemotherapy in the field of colorectal cancer surgery.

The impact of anastomotic leakage on long-term survival has previously been reported for malignant tumours [28–30]. Postoperative anastomotic leakage occurred in 3.7% of our patients. This result is lower compared with the results published by previous studies ([28, 29] 4–20%). One explanation may include the fact that 40% of our patients had a temporary faecal diversion. The relatively low rate of anastomotic leakage in our study does not allow providing any robust conclusions on the relationship between the occurrence of POCs and survival.

Our study has several limitations. One the main limitation includes the single centre design and its retrospective nature which might decrease the ability to generalize the results. A second limitation of our analysis is the relatively short median follow-up time of 42 months. The main strength of this study is that we provide unique and comprehensive insight into the association between the most frequent complications after surgery for T4 CRC and short- and long-term outcomes.

This study provides oncologists additional data that can be used to give patient information to some extent regarding the impact of potentially postoperative complications on long-term survival after T4 CRC surgery. The development of minimally invasive approach might open the door to reduce postoperative complications and time to adjuvant chemotherapy in future studies.

## Conclusion

In conclusion, this study provides persuasive evidence that POCs do not affect the oncological outcomes in patients after the resection of T4 CRC, whether the patient did or did not have synchronous liver metastases, possibly because the prognostic value of the tumour stage in T4 CCR tumours is so important that the corresponding value of POCs becomes negligible [22, 28–49].

## Additional file

Additional file 1: **Table S1.** Additional organs resected with T4 colorectal cancer. (DOCX 17 kb)

## Abbreviations

CRC: Colorectal cancer
CT: Computed tomography
MRI: Magnetic resonance imaging
OS: Overall survival
POC: Post-operative complications
RFS: Recurrence-free survival
