# Supplementary material for: Impact of postoperative complications on long-term survival following surgery for T4 colorectal cancer
Source: BMC Surg. 2018 Oct 17;18:87. doi: 10.1186/s12893-018-0419-y (PMC6192193; doi:10.1186/s12893-018-0419-y)
Supplement: Supplementary file 1 — Table S1. Additional organs resected with T4 colorectal cancer. (DOCX 17 kb) [file 12893_2018_419_MOESM1_ESM.docx]

|  | |
| --- | --- |
| Table S1. Additional organs resected with T4 colorectal cancer | |
| Liver | 11 (27%) |
| Duodenum-pancreas | 1 (2.5%) |
| Small bowel | 12 (30%) |
| Abdominal wall | 2 (5%) |
| Uterus | 4 (10%) |
| Ovaries | 5 (12.5%) |
| Duodenum | 2 (5%) |
| Appendix | 2 (5%) |
| Bladder | 9 (22.5%) |
| Prostate | 1 (2.5%) |
| Stomach | 1 (2.5%) |
| Vagina | 1 (2.5%) |
| Some patients may have had multiple organs resected. | |
